# Endo-1,3(4)-β-Glucanase-Treatment of Oat β-Glucan Enhances Fermentability by Infant Fecal Microbiota, Stimulates Dectin-1 Activation and Attenuates Inflammatory Responses in Immature Dendritic Cells

**DOI:** 10.3390/nu12061660

**Published:** 2020-06-03

**Authors:** Renate Akkerman, Madelon J. Logtenberg, Ran An, Marco Alexander Van Den Berg, Bart J. de Haan, Marijke M. Faas, Erwin Zoetendal, Paul de Vos, Henk A. Schols

**Affiliations:** 1 Immunoendocrinology, Division of Medical Biology, Department of Pathology and Medical Biology, University of Groningen and University Medical Centre Groningen, Groningen, Hanzeplein 1, 9700 RB Groningen, The Netherlands; b.j.de.haan@umcg.nl (B.J.d.H.); m.m.faas@umcg.nl (M.M.F.); p.de.vos@umcg.nl (P.d.V.); 2 Laboratory of Food Chemistry, Wageningen University & Research, Bornse Weilanden 9, 6708 WG Wageningen, The Netherlands; henk.schols@wur.nl; 3 Laboratory of Microbiology, Wageningen University & Research, Stippeneng 4, 6708 WE Wageningen, The Netherlands; ran.an@wur.nl (R.A.); Erwin.Zoetendal@wur.nl (E.Z.); 4 DSM Biotechnology Center, Alexander Fleminglaan 1, 2613 AX Delft, The Netherlands; marco.berg-van-den@dsm.com

**Keywords:** cytokine production, dendritic cells, infant formula, in vitro fermentation, microbiota, oat β-glucan

## Abstract

*Background*: Non-digestible carbohydrates are added to infant formula to mimic the effects of human milk oligosaccharide by acting as prebiotics and stimulating the immune system. Although not yet used in infant formulas, β-glucans are known to have beneficial health effects, and are therefore of potential interest for supplementation. *Methods and results*: We investigated the in vitro fermentation of native and endo-1,3(4)-β-glucanase-treated oat β-glucan using pooled fecal inocula of 2- and 8-week-old infants. While native oat β-glucan was not utilized, both inocula specifically utilized oat β-glucan oligomers containing β(1→4)-linkages formed upon enzyme treatment. The fermentation rate was highest in the fecal microbiota of 2-week-old infants, and correlated with a high lactate production. Fermentation of media supplemented with native and enzyme-treated oat β-glucans increased the relative abundance of *Enterococcus* and attenuated pro-inflammatory cytokine production (IL-1β, IL-6, TNFα) in immature dendritic cells. This attenuating effect was more pronounced after enzyme treatment. This attenuation might result from the enhanced ability of fermented oat β-glucan to stimulate Dectin-1 receptors. *Conclusion*: Our findings demonstrate that endo-1,3(4)-β-glucanase treatment enhances the fermentability of oat β-glucan and attenuates pro-inflammatory responses. Hence, this study shows that especially enzyme-treated oat β-glucans have a high potential for supplementation of infant formula.

## 1. Introduction

Early life nutrition is crucial for life-long health, and strongly influences the development of the innate immune system and the composition of the intestinal microbiota [1,2]. The World Health Organization recommends exclusive breastfeeding for infants up to an age of 6 months [3]. Human milk oligosaccharides (HMOs) are an important component of mother milk which are considered to be prebiotics that guide immune development and gut barrier maturation [4,5]. However, for many reasons, in Europe, only approximately 25% of infants receive exclusive breastfeeding up to an age of 6 months [6]. For infants for which breast milk is not an option, different types of infant formula are commercially available, which are often supplemented with non-digestible carbohydrates (NDCs) to mimic the effects of HMOs. Commonly used NDCs in infant formula include fructo-oligosaccharides (FOS) and galacto-oligosaccharides (GOS), which are added either individually or in combination [7,8]. The beneficial effects of FOS and GOS have been studied extensively [7,8,9]. However, there are many other potential NDCs with beneficial effects on the microbiota composition and the immune system, presumably by simulating the conformational structure of HMOs rather than the constituent monosaccharides [10]. An example of such NDC that could be of interest as a supplement in infant formulas is β-glucan [11].

β-glucans are polymers of D-glucose building blocks linked by β(1→3), β(1→6) or β(1→4) linkages [12], and can be found in many food and feed components including cereals, yeast and mushrooms [13]. β-glucans from cereals, like oat and barley, are linear β(1→3) and β(1→4) linked glucose polysaccharides, whereas β-glucans from yeast and fungi consist of a β(1→3) linked glucan backbone with β(1→6) linked glucose residues as sidechains [12]. Even though β-glucans are comprised of different building blocks than HMOs, e.g., β-linked glucose moieties rather than β-linked glucose, galactose and N-acetylglucosamine that form the backbone of HMOs, β-glucans have been shown to partially resemble the immune modulating functions of HMOs. These effects are exerted via binding to different pattern recognition receptors (PRRs) including C-type lectin Dectin-1, complement receptor 3 and Toll-like receptors (TLRs) [14,15,16]. For oat β-glucans, it has also been shown that microbiota-derived enzymes may enhance their immune activity upon digestion by increasing Dectin-1 stimulation. This was demonstrated by the ability of oat β-glucans to stimulate Dectin-1 receptors, which increased after enzyme treatment with endoglucanase [17].

Next to their direct effects, β-glucans might also influence immunity by changing the composition of the gut microbiota and the formation of short chain fatty acids (SCFAs) [18]. Previous studies demonstrated that the fermentability of β-glucans is dependent on their structure. Enzymatic pretreatment of barley β-glucans enhanced their fermentability by adult fecal microbiota in an in vitro setup [18]. Depolymerization of barley β-glucans by acid hydrolysis into fractions with MWs ranging from 6–104 kDa has also been shown to increase glucan fermentability with the inoculum of 9–15 month-old infants [19]. However, currently, only limited knowledge is available about the effects of fermentation of β-glucans on their immune-modulating properties, and whether an increase in fermentability, due to depolymerization, affects these processes.

In the present study, native oat β-glucan was treated with an enzyme preparation containing predominantly endo-1,3(4)-β-glucanase to decrease the molecular weight. Both native and enzyme-treated oat β-glucan were fermented in an in vitro set-up using the infant fecal inoculum of 2- and 8-week old infants. We decided to use the fecal inoculum of 2- and 8-week-old infants because microbiota compositions are rapidly developing in the first weeks of life [20] and different sets of enzymes for the degradation of NDCs will become available, depending on the bacteria present in the infant gut. Therefore, it is likely that both age classes will have different fermentation capabilities. The glucan degradation kinetics, impact on microbiota composition and SCFA production during fermentation were studied. In addition, the effects of the fermentation digesta on Dectin-1 receptor binding and immature dendritic cells cytokine production were studied.

## 2. Materials and Methods

### 2.1. Oat β-Glucans and Enzymatic Modification

All Native oat β-glucan (OBG) and enzyme-treated oat β-glucan (eOBG) were kindly provided by DSM (Delft, The Netherlands). Native oat β-glucan (medium viscosity, Megazyme, Bray, Ireland) was treated with the commercial enzyme preparation Filtrase NL liquid (DSM batch 614308301), predominantly containing endo-1,3(4)-β-glucanase, to obtain enzyme-treated oat β-glucan. First, native oat β-glucan (5 mg/mL) was dissolved in 10 mM sodium acetate buffer (pH 5.0), after which Filtrase NL liquid was added at 250 µL enzyme/g native oat β-glucan. The mixture was incubated at 37 °C for 5 h in a head-over-tail rotator. Subsequently, enzymes were inactivated by boiling the mixture for 10 min. The final solution was lyophilized for further use. OBG was treated similarly, but without addition of Filtrase NL liquid.

### 2.2. Fermentation of Native and Enzymatic-Treated Oat β-Glucans by Infant Fecal Inoculum

#### 2.2.1. Culture Medium

Standard ileal efflux medium (SIEM; Tritium Microbiology, Veldhoven, The Netherlands) was prepared as described elsewhere with minor modifications [21]. A low amount of carbohydrates was added to mimic the infant ileal environment while minimizing background fermentation. The carbohydrate medium component contained the following carbohydrates in the same ratio: pectin, xylan, arabinogalactan, amylopectin and starch, with a concentration of only 0.24 g/L in the SIEM medium. The pH was adjusted to 5.8 using MES buffer.

#### 2.2.2. Infant Fecal Inoculum

Fecal samples were collected from four exclusively breast-fed and vaginally born infants. The infants did not receive antibiotic treatment and did not have health issues. At the age of 2 and 8 weeks, fecal material was collected from the diaper directly after defecation, transferred to tubes and stored at −20 °C. After the second collection time point, all samples were stored at −80 °C.

The inoculum was prepared as reported elsewhere with some minor modifications [22]. After thawing, the fecal material of four infants was combined (4 × 0.1 g) and diluted in 24 mL sterilized NaCl solution (0.9% (w/v)) in an anaerobic chamber (gas phase: 81% N2, 15% CO2 and 4% H2) (Bactron 300, Sheldon Manufacturing, Cornelius, NC, USA). Homogenization was performed by the addition of sterile glass beads prior to thorough mixing (2000 rpm). After removal of the glass beads, the fecal solution was combined with SIEM medium in a ratio of 5:82 (v/v) and used as pooled fecal inoculum, whose bacterial functionality was found to be largely representative of the fecal inocula of the infant population in general [23].

#### 2.2.3. In Vitro Fermentation

Fermentations were performed in duplicate in an anaerobic chamber. The pooled fecal inoculum was combined with SIEM medium containing native or enzyme-treated oat β-glucan (10 mg/mL) in sterile fermentation flasks at a ratio of 1:10 (v/v). Fermentation flasks were closed with a rubber stopper that was secured with a metal lid to ensure anoxic conditions. Afterwards, flasks were put in an incubator shaker (Innova 40) (37 °C, 100 rpm). At the start and after 14, 20 and 26 h, digesta were collected in triplicate with a syringe. One sample was immediately frozen in liquid nitrogen and stored at −80 °C to preserve the bacteria for later microbial analysis. Both of the other samples were heated for 5 min in a water bath (100 °C) to inactivate the enzymes present. Subsequently, they were stored at −20 °C until further analysis.

The following control fermentations were included: (1) inoculum without glucan substrate to monitor background fermentation, (2) native oat β-glucan and enzyme-treated oat β-glucan without inoculum to monitor contamination.

### 2.3. Fate of OBG and eOBG Upon Fermentation

#### 2.3.1. High Performance Size Exclusion Chromatography (HPSEC)

The degradation of OBG during fermentation was analyzed by HPSEC on an Ultimate 3000 HPLC system (Dionex, Sunnyvale, CA, USA) equipped with a Shodex RI-101 refractive index detector (Showa Denko, Tokyo, Japan). Fermentation samples were diluted to a concentration of 2 mg/mL and centrifuged (5 min, 15,000× *g*). Ten microliters of sample was injected into three TSK-Gel columns connected in series (4000-3000-2500 SuperAW; 150 × 6 mm), preceded by a TSK Super AW-L guard column (35 × 4.6 mm) (Tosoh Bioscience, Tokyo, Japan). The columns covered a molecular mass range from 0–250 kDa. Samples were eluted with 0.2 M NaNO3, at 55 °C with a flow rate of 0.6 mL/min. Pullulan standards (Polymer Laboratories, Palo Alto, CA, USA) were used for calibration.

#### 2.3.2. UHPLC-PGC-MS

To be able to study the fate of specific isomers with different degrees of polymerization (DP), enzyme-treated oat β-glucan was also analyzed on a Vanquish Ultra High Performance Liquid Chromatography (UHPLC) system (Thermo Scientific, San Jose, CA, USA). Prior to analysis, samples were reduced using sodium borohydride to avoid the anomerization of oligosaccharides, followed by a purification step using solid phase extraction [24].

Samples (0.5 µL, 0.5 mg/mL) were injected on a porous graphitic carbon (PGC) column (3 µm particle size, 2.1 mm × 150 mm; Hypercarb, Thermo Scientific) in combination with a guard column (3 µm particle size, 2 mm × 10 mm; Hypercarb, Thermo Scientific). As mobile phase A: ULC-MS water + 0.1% (v/v) formic acid was used. Mobile phase B consisted of ACN + 0.1% (v/v) formic acid. The flow rate was 300 µL/min. The gradient was as follows: 0–2 min, 3% B; 2–21 min, 3–33.6% B; 21–22.5 min, 33.6–100% B; 22.5–30.2 min, 100% B; 30.2–31.7 min, 100–3% B; 31.7–39.4 min, 3% B. The temperature of the autosampler and column oven was controlled at 10 and 25 °C, respectively. A needle wash solvent containing 3% ACN was used to wash the autosampler.

To obtain mass spectrometric (MS) data, the flow of the UHPLC was directed to a Thermo Scientific LTQ-Velos Pro equipped with an electrospray ionization (ESI) probe. Helium and nitrogen were used as sheath and auxiliary gas, respectively. The MS settings were set to a source voltage of 3.5 kV, a source heater temperature of 225 °C, a capillary temperature of 350 °C, a sheath gas flow of 38 and an auxiliary gas flow of 11. MS data in negative mode were collected over a m/z range of 300–2000. A data-dependent MS2 analysis was performed with a normalized collision energy of 35%, activation Q of 0.25, activation time of 10 ms and isolation width of m/z 1.5. MS2 fragmentation was performed on the 1st and 2nd most abundant ion in the MS chromatogram from a parent list containing m/z of reduced enzyme-treated oat β-glucan oligomers. MS3 fragmentation was performed on the product ions with m/z 505 (trimer) and 667 (tetramer). Cellobiose (Sigma-Aldrich, St. Louis, MO, USA), cellotriose (Megazyme, Bray, Ireland) and 1,3:1,4-β-glucotriose (Megazyme) were used as standards for the identification of oat β-glucan oligomers. The relative abundance of oligomers was determined by the selection of the specific mass range followed by the integration of the peaks. Data acquisition and processing were performed using Xcalibur (version 2.2, Thermo Scientific).

### 2.4. Production of SCFAs and Other Organic Acids Upon Fermentation

In order to quantify the production of SCFAs and organic acids, fermentation samples were subjected to GC and HPLC analysis as described elsewhere [25], with minor modifications with respect to the GC analysis. For GC, fermentation samples (1 mg/mL) were mixed in a 2:1 ratio with a solution containing HCl (0.3 M), oxalic acid (0.09 M) and internal standard 2-ethyl butyric acid (0.45 mg/mL). The mixture was allowed to stand at room temperature for 30 min. The temperature profile during GC analysis was as follows: 100 °C, maintained for 0.5 min; raised to 180 °C at 8 °C/min, maintained for 1 min; raised to 200 °C at 20 °C/min, maintained for 5 min. Glass wool was inserted in the glass liner of the split injection port to protect the column from contamination [26].

### 2.5. Microbial Composition Analysis

DNA extraction was performed using Repeated Bead Beating [27], followed by purification using the Maxwell 16 Tissue LEV Total RNA Purification Kit Cartridge (XAS1220) (Promega, Madison, WI, USA). PCR amplification of the V5–V6 region of 16S ribosomal RNA (rRNA) genes using the unique barcoded primer pair BSF784 (RGGATTAGATACCC) and R1064 (CGACRRCCATGCANACCT) was performed in triplicate, as described elsewhere [28] with minor modification. Specifically, for t0 samples which contained 1.4–4.6 ng/μL DNA, a 10 μL DNA template was used for the amplification, correspondingly decreasing the amount of nuclease free water to retain the same reaction volume. For samples with higher DNA concentrations, 0.7 μL of DNA template was used. Two synthetic microbial communities were included as positive controls [29].

After amplification, PCR products were purified using HighPrep PCR kit (MagBio Genomics, Alphen aan den Rijn, The Netherlands). Purified amplicons were quantified using a Qubit dsDNA BR assay kit (Life Technologies, Leusden, The Netherlands). Seventy unique barcode tags were used in each library [29]. Two amplicon pools were formed by combining 200 ng of each barcoded sample, and afterwards concentrated to 50 μL using the HighPrep PCR kit. Libraries were sent for adapter ligation and sequencing with Illumina Hiseq2500 (GATC-Biotech, Konstanz, Germany).

Processing and analysis of the 16S rRNA gene amplicon sequencing data was carried out using the NG-Tax 2.0 pipeline, with default settings and R version 3.5.0 [30]. Amplicon sequencing variants (ASVs) with a relative abundance below 0.1% were removed. The threshold for taxonomic assignment was set at 80%.

Enterococci were analyzed at the level of individual sequences (Amplicon Sequencing Variants (ASVs)). SILVA database release 132 [31] was used for taxonomic classification.

### 2.6. Stimulation of DCs with Oat β-Glucan Fermentation Samples

#### 2.6.1. Cell Culture and Stimulation

Dendritic cells (DCs) generated from umbilical cord blood CD34+ progenitor cells were purchased from the MatTek Corporation (Ashland, MA, USA). Cells were thawed and seeded at a density of 70 × 10^4^ cells/well into 96-well plates and cultured (for 24 h 37 °C, 21% O2 and 5% CO_2_) according to manufacturer’s instructions. After 24 h of culturing, the cells were stimulated withfermentation products of the in vitro batch fermentations of the medium supplemented with native oat β-glucan and enzyme-treated oat β-glucan using infant fecal inoculum. To this end, bacteria were removed from the fermentation samples by centrifugation (10 min, 12,000× *g*). Subsequently, supernatants were filtered through a 0.2 μm filter and diluted in DC-MM culture medium (MatTek Corporation,) containing Polymyxin-B (50 ng/mL) (Invivogen, Toulouse, France) at a ratio of 1:10. The pH was set to 7.4 by the addition of 2M NaOH. DCs were then incubated with 200 μL/well medium containing the fermentation samples for 48 h. After incubation, supernatants were collected and stored at −20 °C until further analysis. All experiments were repeated six times.

#### 2.6.2. Assessment of Cytokine Expression

The levels of MCP-1/CCL2, MIP-1α/CCL3, IL-1β, IL-6, IL-10 and TNFα in the DC supernatant were quantified using a magnetic Luminex^®^ Assay (R&D systems, Biotechne, Minneapolis, MN, USA) according to the manufacturers protocol. The plate was analyzed using a Luminex 200 System. The data obtained was analyzed using the Luminex xPONENT software.

### 2.7. HEK-Dectin1 Reporter Cell Assays

HEK-Blue™ Null1 cells (Invivogen) were stably transfected with pUNO1- hDectin1a or pUNO1-hDectin1b plasmids (InvivoGen) to obtain HEK-Dectin1a and HEK-Dectin1b responsive reporter cells as previously described [32]. HEK-Dectin1 cell lines were cultured and maintained in DMEM culture medium (Lonza, Bornem, Belgium) containing 10% de-activated fetal calf serum (60 °C for 30 min), 50 U/mL Penicillin and 50 μg/mL Streptomycin (Sigma-Aldrich), 100 μg/mL Normocin (Invivogen) supplemented with 12 μg/mL Blasticidin (for HEK-Dectin1a) and 100 μg/mL zeocin (for HEK-Dectin1b) (Invivogen). Both cell lines express soluble embryonic alkaline phosphatase (SEAP) under the control of a NF-κB AP-1 responsive promotor. Stimulation of the Dectin1a or Dectin1b receptor by a Dectin-1 agonist will activate the receptor, and subsequently, the NF-κB transcription factor will be transported to the nucleus, inducing transcription of the reporter gene, resulting in SEAP release into the medium. SEAP can be quantified using Quanti-Blue™ (Invivogen).

HEK-Dectin1a and HEK-Dectin1b cells were seeded into 96-well plates at a density of 10 × 10^4^ cells/well in 200 μL medium. After 24 h, cells were attached and the medium was replaced with fresh medium containing the samples. Fermentation samples of medium supplemented with native oat β-glucan and enzyme-treated oat β-glucan were diluted at a ratio of 1:20 in the culture medium, and the pH was neutralized by the addition of 2M NaOH. As a positive control, 200 μg/mL Zymosan depleted (Invivogen), a particulate β(1→3) glucan with a mean molecular weight of 240 kDa obtained from *Saccharomyces cerevisiae*, was used. Medium was used as a negative control. All samples were incubated for 24 h. Subsequently, the supernatant of activated reporter cells was mixed with Quanti-Blue™ at a ratio of 1:10, and quantified at 650 nm using a Benchmark Plus Microplate Reader using Microplate Manager 5.2.1 version for data acquisition. Experiments were repeated three times in triplicate.

### 2.8. Statistical Analysis

Data were analyzed using Prism 8 software (GraphPad, San Diego, CA, USA). For the cytokine data, outliers were removed after testing using a Grubbs outlier test (alpha = 0.05). Cytokine data are shown as average with standard error of the mean (SEM). Data were distributed normally, and analyzed using a mixed-effects model (REML) to test for differences between the different samples, followed by a Tukey’s multiple comparisons test to compare cytokine responses between different groups. *p*-values < 0.05 were considered significant. Data from HEK-Dectin1 stimulation experiments were tested using a OneWay ANOVA test followed by a Tukey’s multiple comparison test. *p*-values < 0.05 were considered significant.

## 3. Results

### 3.1. Formation of Oligomers Upon Endo-1,3(4)-β-Glucanase Treatment of Native Oat β-Glucan

To determine whether a decrease in molecular weight would impact the fermentability and immune modulating capacity, native oat β-glucan (300 kDa) was treated with a commercial enzyme preparation which predominantly contained endo-1,3(4)-β-glucanase. The enzyme treatment of native oat β-glucan was monitored with UHPLC-PGC-MS, and resulted in the formation of oligomers with a DP ranging from 2–5 (Table S1). The different oligomers were characterized based on MS2 and MS3 fragmentation and available references. Cellobiose (2a), cellotriose (3a), cellobiosyl-(1→3)-β-d-cellobiose (3b), glycosyl-(1→3)-β-d-cellotriose (4a) and a partially characterized DP5 oligomer (5a) were recognized to be present (Table S1), with glucosyl-(1→3)-β-d-cellotriose being most abundant (58%).

### 3.2. Size- and Linkage-Specific Fermentation of Oat β-Glucan (Oligomers) by Infant Fecal Microbiota

Since the microbiota composition and functionality depend on the age of the infant [33], the degradation of native and enzyme-treated oat β-glucan was studied using the fecal microbiota of 2- and 8-week-old infants. Fermentation digesta were collected at 0, 14 and 26 h of in vitro batch fermentation, followed by HPSEC and UHPLC-PGC-MS analysis to monitor the fermentability of the native and enzyme-treated oat β-glucan, respectively.

**Figure 1:**
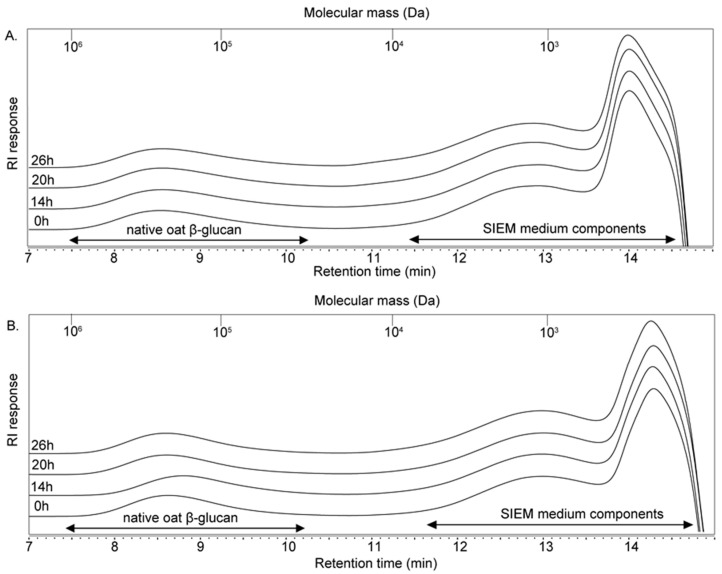
HPSEC profiles of native oat β-glucan at the start and after 14, 20 and 26 h of fermentation using pooled fecal inoculum of 2- (**A**) and 8- (**B**) week-old infants. Calibration of the system using pullulan standards is indicated.

The fecal microbiota of both 2-and 8-week-old infants were unable to degrade native oat β-glucan, as no significant shifts in the molecular weight of native oat β-glucan were observed over 26 h of fermentation (Figure 1).

**Figure 2:**
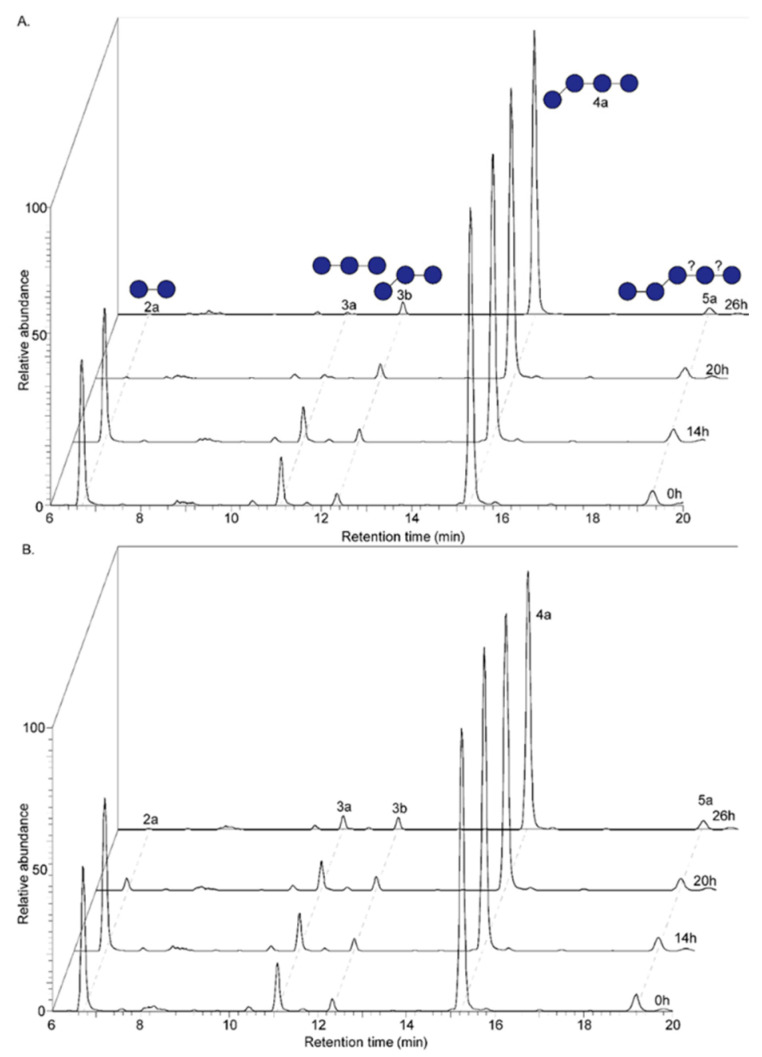
UHPLC-PGC-MS profiles of enzyme-treated oat β-glucan (eOBG) at the start and after 14, 20 and 26 h of fermentation using pooled fecal inoculum of 2- (**A**) and 8- (**B**) week-old infants. Peaks are labelled with 1a: 1 = degree of polymerization, a = letter qualifier. Corresponding structures are placed above label with —: β(1–4)-linkage and /: β(1–3)-linkage.

The treatment of native oat β-glucan with endo-1,3(4)-β-glucanase increased the fermentability to a large extent (Figure 2A and Figure S1). The fecal microbiota of 2-week-old infants were able to almost completely ferment oligomer 2a and 3a, while oligomer 3b and 4a with a β(1→3)-linked glucose at the non-reducing end remained virtually intact. The partially characterized DP5 oligomer (5a) was degraded by 50% after 26 h of fermentation. The increase of oligomer 3b and 4a during fermentation is assumed to be caused by the partial degradation of oligomer 5a. Since the most abundant oligomer, i.e., 4a, was highly resistant to fermentation, 61% of the total mixture remained after 26 h of fermentation.

The fecal microbiota of 8-week-old infants showed a similar preference for oligomers of enzyme-treated oat β-glucan which consisted solely of β(1→4)-linked glucosyl residues (Figure 2B and Figure S1). However, the rate of fermentation was lower compared to the fecal microbiota of 2-week-old infants, as demonstrated by oligomer 3a and 5a, or which 32% and 60% remained, respectively, after 26 h of fermentation. Nonetheless, in comparison to the non-fermentable native oat β-glucan, the enzyme treatment of native oat β-glucan increased the fermentability significantly for both 2- and 8-week-old infants.

### 3.3. Increase in Enterococcus during Fermentation of Media Supplemented with Native and Enzyme-Treated Oat β-Glucan

**Figure 3:**
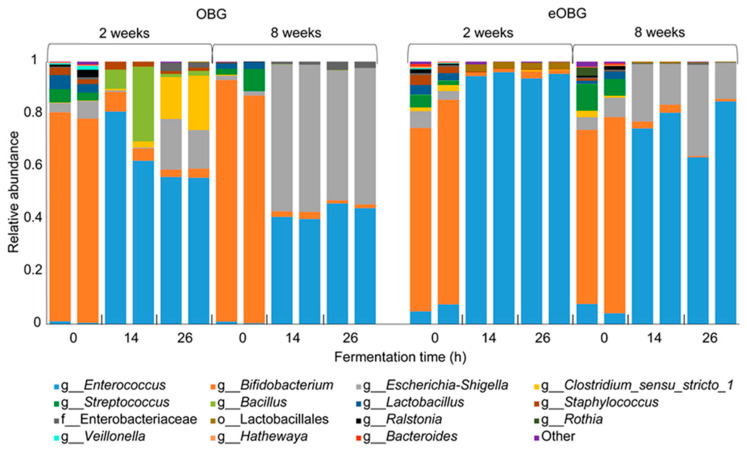
Relative abundance of bacteria at the highest classified taxonomy in duplicate fermentation digesta collected at the start and after 14 and 26 h from in vitro fermentation of media supplemented with native oat β-glucan (OBG) and enzyme-treated oat β-glucan (eOBG) using pooled fecal inoculum of 2- and 8-week-old infants.

To study the impact of native and enzyme-treated oat β-glucan on infant microbiota composition, 16S rRNA gene amplicon sequencing was performed. The fermentation of media supplemented with native oat β-glucan increased the relative abundance of *Enterococcus* in the fecal inoculum of 2-week-old infants (Figure 3). After 14 h of fermentation, ~72% of the bacteria belonged to the genus *Enterococcus*, which decreased slightly to a ~56% relative abundance after 26 h of fermentation. Since the fermentation control without the addition of native oat β-glucan showed a less pronounced increase in *Enterococcus* for the fecal inoculum of 2-week-old infants (~26% after 26 h) (Figure S2), it is suggested that the growth of *Enterococcus* was stimulated by the presence of the unfermentable native oat β-glucan. Next, an increase in *Bacillus* up to ~18% was observed in the first 14 h of fermentation of media supplemented with native oat β-glucan, which decreased to ~1% after 26 h of fermentation (Figure 3). In contrast, a clear increase in relative abundance was observed for both *Escherichia-Shigella* and *Clostridium Sensu Stricto I*, up to ~17 and ~18% respectively, after 26 h of fermentation. However, this increase was similar for *Clostridium Sensu Stricto I*, and even more pronounced for *Escherichia-Shigella* in a control fermentation without native oat β-glucan (Figure S2). As these genera have been reported to grow well on peptides and amino acids which are highly abundant in SIEM medium [34], it cannot be excluded that their growth was stimulated by SIEM medium components.

The fermentation of media supplemented with native oat β-glucan by fecal microbiota of 8-week-old infants induced therelative abundance of *Enterococcus*, although to a lesser extent than with the inocula of 2-week-old infants (Figure 3). After 14 h of fermentation, ~40% of the bacteria belonged to the genus Enterococcus, which increased slightly, i.e., up to ~45%, between 14 and 26 h of fermentation. In contrast to 2-week-old infants, no increase in the relative abundance of *Bacillus* and *Clostridium Sensu Stricto I* were observed. However, similar background fermentation of SIEM medium components by *Escherichia-Shigella* was observed, contributing to ~50% of all bacteria after 26 h of fermentation.

The fermentation of enzyme-treated oat β-glucan resulted in a higher increase in the relative abundance of *Enterococcus*, as was observed for the fermentation of media supplemented with native oat β-glucan. After 14 and 26 h of fermentation by the fecal microbiota of 2-week-old infants, more than 94% of the bacteria belonged to the genus *Enterococcus*. The increase in *Enterococcus* was less pronounced with 8-week-old infants, as the relative abundance increased to ~75% after both 14 and 26 h of fermentation. Similar to the fermentation of media supplemented with native oat β-glucan, background fermentation of SIEM medium components by *Escherichia-Shigella* was observed for the inoculum from 8-week-old-infants.

### 3.4. Increase of Specific Enterococcus ASVs during Fermentation of Media Supplemented with Native and Enzyme-Treated Oat β-Glucan is Dependent on Infant Age

Since the relative abundance of *Enterococcus* after fermentation of media supplemented with native and enzyme-treated oat β-glucan was higher with fecal microbiota of 2-week-old infants than with fecal microbiota of 8-week-old infants, we questioned whether this might be explained by differences in *Enterococcus* species present. To this end, *Enterococcus* Amplicon Sequencing Variants (ASVs) were examined, which can be considered as a proxy for *Enterococcus* species. A difference in *Enterococcus* ASVs was observed between the fermentations using both inocula (Figure S3), which was most pronounced for enzyme-treated oat β-glucan. After 26 h of fermentation of enzyme-treated oat β-glucan, ASV 39703838 was most abundant in the fecal inoculum of 2-week-old infants, contributing to ~81% of the enterococci. With the fecal inoculum of 8-week-old infants, ASV 39703836 was most abundant, contributing to ~89% of the enterococci. A comparison of the sequences of ASV 39703838 and 39703836 (Table S2) with the SILVA database release 132 showed an exact match with *Enterococcus faecium* and *Enterococcus faecalis,* respectively.

### 3.5. Lactate, a Key Metabolite Formed during Enzyme-Treated Oat β-Glucan Fermentation

**Figure 4:**
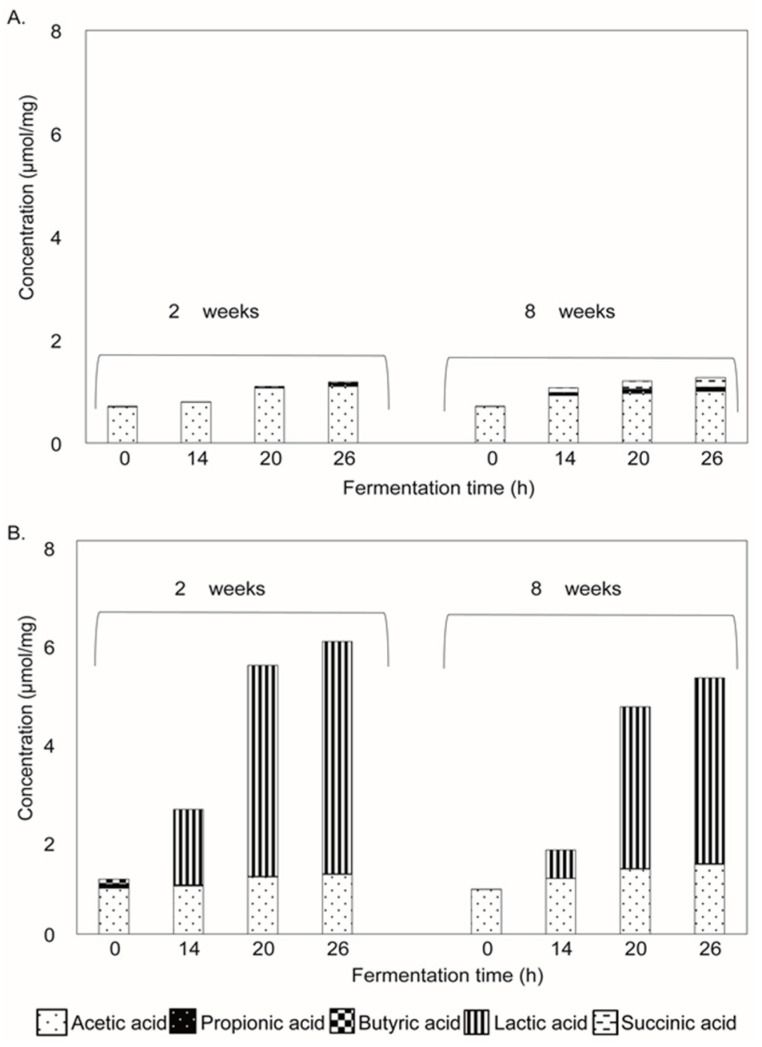
Production of SCFAs, lactic acid and succinic acid upon fermentation of media supplemented with native oat β-glucan (**A**) and enzyme-treated oat β-glucan (**B**) using pooled fecal inoculum of 2- and 8-week-old infants.

The production of SCFAs and organic acids during the fermentation of media supplemented with native oat β-glucan and enzyme-treated oat β-glucan was studied, as these metabolites have been shown to have important immunomodulatory activity [35,36]. The digesta collected during fermentation were analyzed by GC and HPLC to quantify the SCFAs and other organic acids (Figure 4). The organic acids present at the start of the fermentation originated from the sodium acetate buffer used in the preparation of the oat β-glucans, which was confirmed by the detection of a similar level of organic acids in the control fermentations without added inoculum (Figure S4). Reproducibility was confirmed with duplicate fermentations.

The fermentation of media supplemented with native oat β-glucan by both inocula resulted in a negligible total organic acid production, i.e., 1.2–1.3 µmol/mg after 26 h (Figure 4), compared to the control fermentations without added oat β-glucan (~1 µmol/mg; Figure S4). The enzymatic treatment of native oat β-glucan increased the production of organic acids (Figure 4). Fermentation by fecal microbiota of 2-week-old infants resulted in a total level of 2.5 µmol/mg after 14 h of fermentation, which increased to 6 µmol/mg after 26 h. Lactate, acetate, butyrate and propionate were present in a ratio of 79.4:20.4:0.1:0.1. The production of organic acids was slightly lower in the fecal microbiota of 8-week-old infants (Figure 4). After 14 h of fermentation of enzyme-treated oat β-glucan, a total level of 1.8 µmol/mg was reached, which increased up to 5.2 µmol/mg after 26 h of fermentation. Lactate, acetate, butyrate and propionate were present in a ratio of 72.4:27.3:0.2:0.1.

### 3.6. Cytokine Production by Dendritic Cells (DC) Differs between Fermentation Digesta of Media Supplemented with Native Oat β-Glucan and Enzyme Treated Oat β-Glucan

DCs are present under the epithelial lining of the gastrointestinal tract, and can therefore come into contact with dietary components such as oat β-glucan and its fermentation products. To test whether the digesta of media supplemented with native oat β-glucan and enzyme-treated oat β-glucan fermented with the fecal inoculum of 2- and 8-week-old infants affects intestinal immune responses, we incubated DCs from umbilical vein with fermentation samples taken at different time points. The pro-inflammatory chemokines and cytokines MCP-1/CLL2, MIP-1α/CCL3, IL-1β, IL-6 and TNFα, as well as the anti-inflammatory cytokine IL-10 induced in the DC supernatant, were studied.

**Figure 5:**
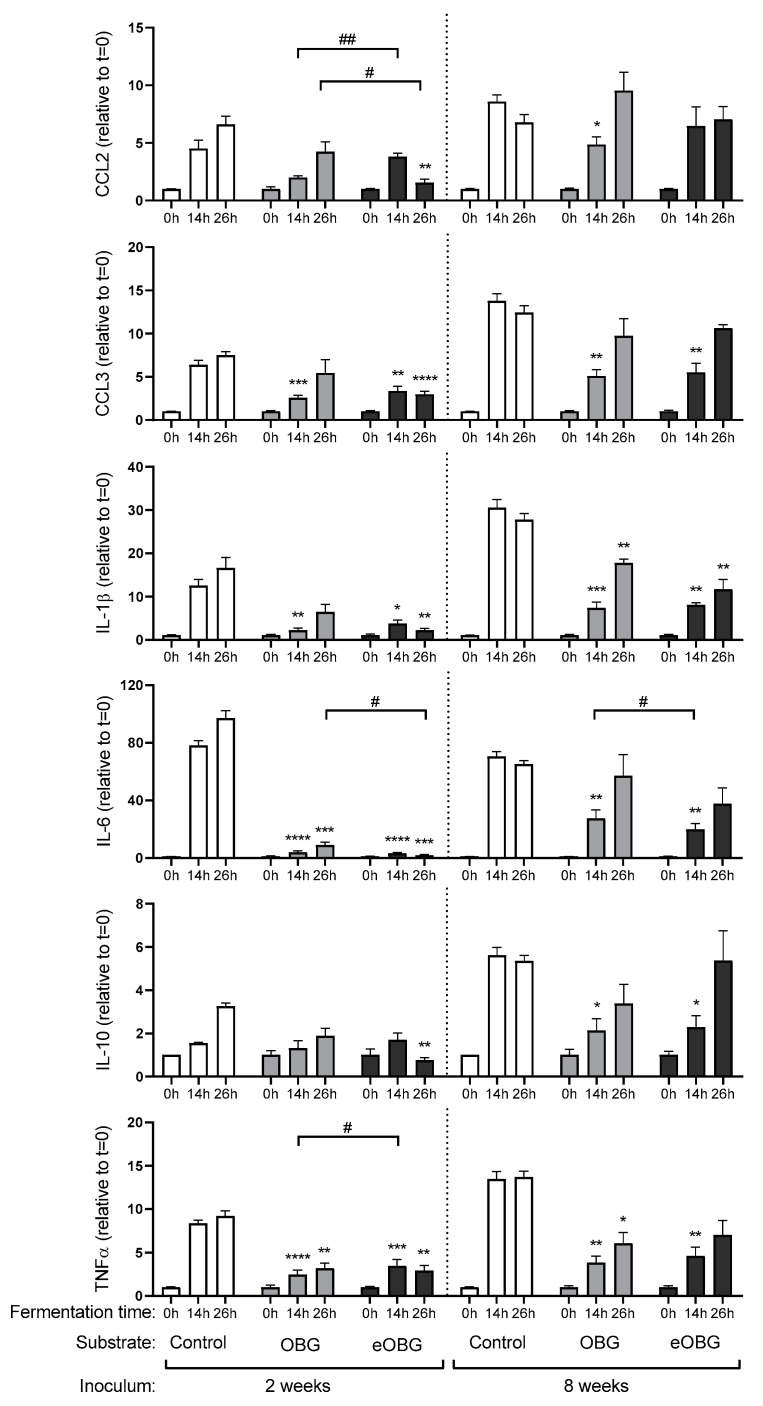
Induced cytokines (fold change of induction by 0 h sample) by digesta of control fermentation, native oat β-glucan (OBG) and enzyme-treated oat β-glucan (eOBG) fermentation with pooled fecal inocula of 2- and 8-week-old infants. Statistical differences were tested using a mixed-effects model (REML) test followed by a Tukey’s multiple comparison test. Stars above bars represent statistical differences compared to the time-matched control samples (* *p* < 0.05, ** *p* < 0.01, *** *p* < 0.001, **** *p* < 0.0001). Statistical differences between samples of OBG and eOBG fermentations are indicated above graphs (# *p* < 0.05, ## *p* < 0.01, ### *p* < 0.001, #### *p* < 0.0001) (*n* = 6).

The digesta from the control fermentation with only inoculum and SIEM medium were incubated with DCs (Figure 5). The digesta of both *t* = 14 and *t* = 26 from fermentations with either fecal inoculum of 2- or 8-week-old infants induced the production of all of the measured chemokines and cytokines. Incubation of DCs with *t* = 14 digesta of media supplemented with native oat β-glucan fermented with inoculum of 2-week-old infants attenuated DC activation and lowered production of MIP1α/CCL3, IL-1β, IL-6 and TNFα, compared to the *t* = 14 digesta of the control fermentation (Figure 5). The incubation with *t* = 26 digesta also induced lower levels of IL-6 and TNFα compared to the control. The *t* = 14 digesta of enzyme-treated oat β-glucan fermented with the fecal inoculum of 2-week-old infants also resulted in lower levels of MIP1α/CCL3, IL-1β, IL-6 and TNFα in DCs, compared to the *t* = 14 digesta of the control fermentation. Also, incubation with *t* = 26 digesta resulted in lower levels of all of the measured chemokines and cytokines compared to the control. When the chemokine and cytokine patterns of DCs stimulated with native oat β-glucan and enzyme-treated oat β-glucan of fermentations with fecal inoculum of 2-week-old infants were compared, it was found that incubation with *t* = 14 digesta of native oat β-glucan fermentation resulted in a significantly lower level of MCP-1/CLL2 and a higher level of TNFα. In addition, the *t* = 26 digest of native oat β-glucan induced a significantly higher level of MCP-1/CLL2 and a lower level of IL-6, compared to the *t* = 26 digesta of enzyme-treated oat β-glucan.

The incubation of DCs with *t* = 14 digesta of native oat β-glucan fermented with fecal inoculum of 8-week-old infants induced a lower level of all of the measured chemokines and cytokines compared to the control. The *t* = 26 digesta reduced the levels of IL-1β and TNFα compared to the control. The *t* = 14 digesta of enzyme-treated oat β-glucan fermented with the fecal inoculum of 8-week-old infants induced significant lower levels of MIP1α/CCL3, IL-1β, IL-6, IL-10 and TNFα, while the *t* = 26 digest of this fermentation only decreased the expression of IL-1β in DCs, compared to the control. When the chemokine and cytokine patterns of DCs stimulated with the native oat β-glucan or enzyme-treated oat β-glucan digesta of fermentations with 8-week inoculum were compared, the *t* = 14 digesta of the native oat β-glucan fermentation induced significant higher IL-6 levels, compared to the *t* = 14 digesta of the enzyme-treated oat β-glucan fermentation.

### 3.7. Fermentation of Media Supplemented with Native Oat β-Glucan and Enzyme-Treated Oat β-Glucan by Infant Fecal Inoculum Enhances Dectin-1 Activation

As Dectin-1 is the most important immune receptor for β-glucans, and is present on DCs [37], we also investigated whether the enzyme treatment of native oat β-glucan and glucan fermentation with the inoculum of 2- and 8-week-old infants would impact the activation of the Dectin-1 receptor. To this end, HEK-Dectin1a and HEK-Dectin1b reporter cell lines were incubated with digesta taken from the fermentation of native oat β-glucan and enzyme-treated oat β-glucan. Normal cell culture medium and Zymosan, a particulate β-glucan which is known to have Dectin-1 stimulating activity, were used as the negative and positive controls, respectively.

**Figure 6:**
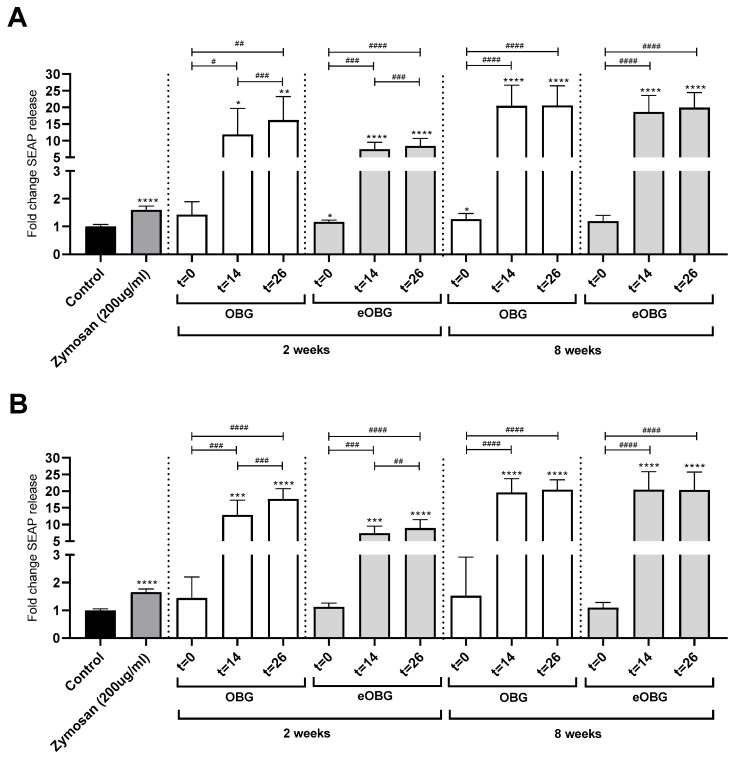
Relative NF-κB release by (**A**) HEK-Dectin1a and (**B**) HEK-Dectin1b reporter cell stimulation with (**A**) samples of native oat β-glucan (OBG) and enzyme-treated oat β-glucan (eOBG) fermented with 2-week old fecal inoculum and (**B**) OBG and eOBG fermented with 8-week old fecal inoculum. For all groups, a medium control and Zymosan were included. Statistical differences were tested using a OneWay ANOVA test followed by a Tukey’s multiple comparison test. Stars above bars represent statistical differences compared to the medium control (* *p* < 0.05, ** *p* < 0.01, *** *p* < 0.001, **** *p* < 0.0001). Statistical differences between *t* = 14 or *t* = 26 and the matched *t* = 0 are indicated above graphs (# *p* < 0.05, ## *p* < 0.01, ### *p* < 0.001, #### *p* < 0.0001). Experiments are repeated three times in triplicate.

First, samples of native oat β-glucan and enzyme-treated oat β-glucan fermented with either 2- or 8-week-old infant fecal inoculum were incubated with the HEK-Dectin1a reporter cells (full length variant) (Figure 6A). The *t* = 0 digesta of enzyme-treated oat β-glucan fermented with the inoculum of 2-week-old infants and the *t* = 0 digesta of native oat β-glucan fermented with the inoculum of 8-week-old infants resulted in slightly higher SEAP release in HEK-Dectin1a reporter cells, compared to the medium control. All *t* = 14 and *t* = 26 of native oat β-glucan and enzyme-treated oat β-glucan fermented with either 2- or 8-week inoculum could induce significantly higher SEAP release in HEK-Dectin1a reporter cells, compared to the medium control and their own *t* = 0 matched digesta. For native oat β-glucan and enzyme-treated oat β-glucan fermented with the inoculum of 2-week-old infants, the Nf-κB release was also higher for *t* = 26 digesta, compared to the *t* = 14 digesta.

Fermentation digesta of native oat β-glucan and enzyme-treated oat β-glucan were also incubated with HEK-Dectin1b reporter cells (stalk-less variant) (Figure 6B). None of the *t* = 0 digesta of native oat β-glucan and enzyme-treated oat β-glucan fermented with either 2- or 8-week inoculum were able to induce significant SEAP release, compared to the medium control. The *t* = 14 and *t* = 26 digesta of native oat β-glucan and enzyme-treated oat β-glucan fermented with the inoculum of either 2- or 8-week-old infants could induce significant higher SEAP release in HEK-Dectin1b reporter cells compared to the medium control. Again, the induction of SEAP release of all of these digesta was also higher, compared to their own *t* = 0 matched digesta. For native oat β-glucan and enzyme-treated oat β-glucan fermented with the inoculum of 2-week old infants, the SEAP release was also higher by *t* = 26 digesta compared to *t* = 14 digesta.

## 4. Discussion

Non-digestible carbohydrates are often added to cow milk-based infant formula to substitute HMO functions. Commonly used NDCs are FOS and GOS; however, β-glucans might also have potential as possible addition to infant formula, as these molecules also stimulate the growth of beneficial microbes [18,19] and have immune modulating effects [15,16]. However, at present, only limited knowledge is available about the effects of fermentation of oat β-glucans on their immune-modulating properties. In this study, the degradation of native and enzyme-treated oat β-glucan by infant fecal microbiota was studied, as well as the effect of the fermentation digesta on cytokine production by dendritic cells and dectin-1 receptor activation, i.e., the receptor for β-glucans on immune cells [37].

Although it has been demonstrated in vitro that adult fecal microbiota can utilize native oat and barley β-glucans in the size range of 130–243 kDa [18], native oat β-glucan was not degraded by the fecal microbiota of either 2- or 8-week-old infants in the present study. This finding, however, is in line with the differences observed in GI microbial functionality in people of different ages [38]. Bacterial species belonging to the *Clostridium histolyticum* group were mainly involved in the described utilization in adults, which was corroborated by the presence of genes encoding endo-β-glucanase for several members of this group, e.g., *C. longisporum* [39] and *C. acetobutylicum* [40]. The stimulation of *Clostridium* was also observed upon intake of barley β-glucans in an in vivo study with healthy adult volunteers [41]. In our study, using infant microbiota, bacteria belonging to the genus *Clostridium sensu stricto 1* comprising many different *Clostridium* species [31] were detected. Nevertheless, the absence of fermentation of native oat β-glucan by infant fecal microbiota could possibly be ascribed to differences at the species level, since the presence and abundance of specific *Clostridium* species gradually changes upon aging [42].

Instead, a clear increase in *Enterococcus* was observed upon fermentation of media supplemented with native oat β-glucan by the fecal microbiota of 2- and 8-week-old infants, which was more pronounced than in the control fermentations without added native oat β-glucan. As such, it is suggested that the presence of the unfermentable native oat β-glucan stimulated the growth of Enterococcus on SIEM medium components. As enterococci are known to be among the first colonizers of the infant gut [43], the increase in enterococci might be relevant in infants for creating a new environment that allows colonization of the gut by strict anaerobes [2,20]. A similar stimulation of *Enterococcus* was shown in an in vitro fermentation study of barley β-glucans using the fecal microbiota of 9–15-month old infants which received both human milk and solid foods [19]. With these age groups, substantial production of SCFAs was also observed, which indicates that the fecal microbiota of 9–15-month-old infants are capable of degrading native oat β-glucan. It is therefore likely that differences in *Enterococcus* on species level upon aging [44,45] result in the availability of different sets of enzymes for the degradation of β-glucans for infants of different age groups. Our findings suggest that the bacteria and their enzymes which are responsible for the degradation of β-glucans are not yet present the first weeks after birth, but are introduced over time.

In contrast to native oat β-glucan that was not fermented by infant fecal microbiota, endo-1,3(4)-β-glucanase-treated oat β-glucan was degraded by the fecal microbiota of both 2- and 8-week old infants. This result corroborates the findings of Lam et al. [19], who observed an increase in SCFA production when lowering the molecular weight of barley β-glucans in an in vitro fermentation study using infant fecal inocula. We also found that decreasing the size of oat β-glucans by enzyme treatment resulted in a stronger stimulation of *Enterococcus* and increased production of lactic acid, which were more pronounced in the fecal inoculum of 2-week-old infants. Our data suggest that the fermentation of enzyme-treated oat β-glucan resulted in a selective increase of *E. faecium* and *E. faecalis* using the fecal inoculum of 2- and 8-week-old infants, respectively. Notably, for both inocula, there was a clear preference for the β(1→4)-linked oligomers present in the enzyme-treated oat β-glucan. Not much is known about specific carbohydrate-degrading enzymes and their structural preferences expressed by *Enterococcus* species. However, a previous in vitro study using adult fecal inoculum also concluded that lower molecular weight barley β-glucans results in the stimulation of *Enterococcus* and *Lactobacillus* [18].

The fermentation digesta of both native and enzyme-treated oat β-glucan could attenuate multiple pro-inflammatory cytokine responses in immature dendritic cells. The digesta of the control fermentation with the inoculum of either 2- or 8-week-old infants induced high levels of the chemokines and cytokines MCP-1/CLL2, MIP-1α/CCL3, IL-1β, IL-6 and TNFα, as well as the anti-inflammatory cytokine, IL-10. This effect was only observed for the *t* = 14 and *t* = 26 digesta, and therefore, was likely caused by products formed during fermentation, like metabolic products of protein fermentation and other bacterial metabolites such as ATP, lipoteichoic acid, polysaccharide A, peptidoglycan, RNA/DNA sequences and exopolysaccharide [46]. The most pronounced attenuating effect by the digesta of medium supplemented with native and enzyme-treated oat β-glucan was observed for IL-6 production. The strongest attenuation of IL-6 was observed for the digesta of fermentations with the fecal inoculum of 2-week-old infants. Incubation with *t* = 14 and *t* = 26 digesta resulted in a significantly lower induction of IL-6 compared to the controls. In addition, the *t* = 26 digesta of enzyme-treated oat β-glucan resulted in a significantly lower IL-6 production compared to the *t* = 26 digesta of native oat β-glucan. The observed difference in DC stimulating capacity could possibly be explained by the formation of lactate which was only observed during the fermentation of enzyme-treated oat β-glucan. Lactate is the main end-product of the fermentation of carbohydrates by Enterococcus [47], and has been reported to modulate cytokine responses, resulting predominantly in reduced inflammation in PBMCs and monocytes [48].

Despite the absence of glucan degradation and lactate formation, the digesta of medium supplemented with native oat β-glucan displayed significant immune attenuating effects. As with the fermentation of both medium supplemented with native and enzyme-treated oat β-glucan, a clear increase in *Enterococcus* was observed; it is suggested that the microbiota composition plays an important role in the attenuation of pro-inflammatory cytokine responses. The unfermentable native oat β-glucan resulted in a lower abundance of Enterococcus after 26 h of fermentation than the fermentable enzyme-treated oat β-glucan. Next to the SCFAs, differences in the relative abundance of *Enterococcus* could thus also possibly explain the difference in DC stimulation capacity between native and enzyme-treated oat β-glucan digesta. Although the bacterial content was removed from the digesta before incubation with DCs in our experiments, the presence of soluble immune-active bacterial fragments in the digesta might be possible, as exemplified for the supernatant of a *Lactobacillus casei* cell wall extract [49].

*Enterococcus* have been associated with immunomodulatory functions and gut health in several studies. For example, a cohort study by Bjorksten et al. found a negative correlation between the colonization of *Enterococcus* in the first year of life and the development of allergies [50]. Other in vivo studies observed a correlation between *E. faecium* and the reduction of infections in the gut, as reviewed by Franz et al. [51]. There are also multiple studies demonstrating the immunomodulatory properties of *E. faecalis* on different cell types. For example, *E. faecalis* could upregulate IL-10 through PPARγ1 activation in colonic cell lines [52]. A study by Wang et al. showed that several strains of *E. faecalis* isolated from newborn infants downregulated IL-8 secretion in Caco-2, HT-29 and HCT116 intestinal cell lines [53]. Interestingly, the immune attenuating effects were virtually gone when the carbohydrate moieties on the cell surface of *E. faecalis* were oxidized. This indicates the carbohydrates present on the cell surface of *E. faecalis* are the major effector molecules for regulating IL-8 secretion [53]. An additional study by Wang et al. demonstrated that IL-8 secretion in intestinal cell lines is attenuated by *E. faecalis* through the inhibition of MAPK signaling pathways. It was suggested that this effect was also caused by factors on the exterior cell wall of the bacteria [54]. As such, it can be hypothesized that the enzyme treatment induced the immune-attenuating effect of native oat ß-glucan due to the increased stimulation of *Enterococcus* and consequent production of lactate during fermentation.

As Dectin-1 receptors are important β-glucan binding receptors present on DCs [55], we tested the immune stimulating capacity of our digesta on Dectin-1 reporter cell lines. The *t* = 0 digesta of medium supplemented with the native and enzyme-treated oat β-glucan did not stimulate the Dectin-1 receptors. This is in line with an earlier study showing that particulate β-glucans induce stronger immune responses than soluble β-glucan [15], as, in our study, we used soluble β-glucan, and any insoluble material was removed from the digesta by centrifugation and filtration prior to incubation with the receptors. The absence of any insoluble material could possibly also explain why we did not observe an increase in Dectin-1 stimulation after endoglucanase treatment, as observed before [17].

Both the Dectin-1a and Dectin-1b receptors were strongly activated by the *t* = 14 and *t* = 26 digesta of both native and enzyme-treated oat β-glucan, fermented with the inoculum of either 2- or 8-week-old infants. It is unlikely that conformational changes of oat β-glucan played a role in the receptor activation, as reported elsewhere [56], as the stimulatory effects of the *t* = 14 and *t* = 26 digesta of the unfermentable native oat β-glucan and the fermented enzyme-treated oat β-glucan were very similar. This finding also indicates that the degradation of the enzyme-treated oat β-glucans and the consequent lactate production were not responsible for the observed effects. It is tempting to speculate that the observed Dectin-1 activation is an (in)direct consequence of the increase in *Enterococcus*. Although it is commonly accepted that fungal β-glucan with β(1→3) glycosidic bonds is an agonist of Dectin-1, there are reports of Dectin-1 activation by β(1→1) trehalose [57], mannoprotein [57], lipopeptide [58] and bacterial cell walls [49,59]. As such, it could be hypothesized that the complex cell wall of *Enterococcus* containing, amongst others, lipoteichoic acid, surface proteins and capsular polysaccharides [60], plays a role in the activation of Dectin-1 receptors, as observed in our study. The activation of Dectin-1 receptors has been reported to have immune effects. For example, in macrophages, Dectin-1 regulates IL-10 and induces regulatory macrophage markers [61]. The ability of the *t* = 14 and *t* = 26 digesta to stimulate Dectin-1 activation, therefore, might contribute to the immune attenuating effects we observed in DCs.

## 5. Conclusions

Our data shows that the fermentation capacity of oat β-glucans by the fecal microbiota of 2- and 8-week-old infants increases after endo-1,3(4)-β-glucanase treatment. Both native and enzyme-treated oat β-glucans can attenuate pro-inflammatory responses, a phenomenon that was more pronounced for enzyme-treated oat β-glucans. It is suggested that this attenuation is caused by the stimulation of *Enterococcus* upon fermentation, and by the increased stimulation of Dectin-1 receptors by the fermentation digesta. Hence, this study shows that especially enzyme-treated oat β-glucans are highly relevant for infant formula, as they exert beneficial effects on both microbiota composition and immunomodulation in infants.

## Supplementary Materials

The following are available online at https://www.mdpi.com/2072-6643/12/6/1660/s1, Figure S1: Percentages of remaining enzyme-treated oat β-glucan oligomers during in vitro fermentation using pooled fecal inoculum of 2- (A) and 8- (B) week-old infants. Concentrations per oligomer in the original enzyme-treated oat β-glucan were set at 100%. Chemical structures of oligomer 2a, 3a, 3b, 4a and 5a are summarized in Table S1. Table S1. Overview of characterized oligomers formed upon enzymatic treatment of oat β-glucans with the relative abundance of the MS2 and MS3 fragments with *m/z* 263 with a: product ion with *m/z* 505 and b: product ion with *m/z* 667. Relative abundance of each component is determined by integration of peak areas in UHPLC-PGC-MS with sum of all as 100%. Figure S2: Relative abundance of bacteria at the highest classified taxonomy in fermentation digesta collected at the start and after 14 and 26 h from in vitro fermentation containing solely SIEM medium and fecal inoculum of 2- and 8-week-old infants. Table S2. Sequences of *Enterococcus* ASVs 39703836 and 39703838. Figure S3: Relative abundance of *Enterococcus* Amplicon Sequencing Variants in duplicate fermentation digesta collected at the start and after 14 and 26 h from in vitro fermentation of media supplemented with native oat β-glucan (OBG) and enzyme-treated oat β-glucan (eOBG) using pooled fecal inoculum of 2- and 8-week-old infants. Figure S4: Production of SCFAs, lactic acid and succinic acid upon in vitro fermentation containing solely native oat β-glucan (OBG) ÷ enzyme-treated oat β-glucan (eOBG) and SIEM medium (A) or infant fecal inoculum and SIEM medium (B). The acetate in the control fermentation without added inoculum is primarily originating from the sodium acetate buffer used in the OBG and eOBG preparation.
