# Supplementary material for: Endo-1,3(4)-β-Glucanase-Treatment of Oat β-Glucan Enhances Fermentability by Infant Fecal Microbiota, Stimulates Dectin-1 Activation and Attenuates Inflammatory Responses in Immature Dendritic Cells
Source: Nutrients. 2020 Jun 3;12(6):1660. doi: 10.3390/nu12061660 (PMC7352905; doi:10.3390/nu12061660)
Supplement: Supplementary file 1 [file nutrients-12-01660-s001.pdf]

Supplementary figures

Table S1. Overview of characterized oligomers formed upon enzymatic treatment of oat β-glucans with the relative abundance of the MS² and MS³ fragments with m/z 263 with [a]: product ion with m/z 505 and [b]: product ion with m/z 667. Relative abundance of each component is determined by integration of peak areas in UHPLC-PGC-MS with sum of all as 100%.

Peak no.	Tr (min)	$[M-H]^-$	Relative abundance (%)	Tentative / Conclusive identification	MS^2 m/z 263 (%)	MS^3 m/z 263 (%)	Confirmation by available reference
2a	6.7	343	27	β-D-Glc-(1→4)-D-Glc	n.d.		+
3a	11.1	505	8	β-D-Glc-(1→4)-βD-Glc-(1→4)-D-Glc	20.8		+
3b	12.4	505	2	β-D-Glc-(1→3)-βD-Glc-(1→4)-D-Glc	0.2		+
4a	15.3	667	58	β-D-Glc-(1→3)-β-D-Glc-(1→4)-β-D-Glc-(1→4)-D-Glc	0.7	8.3[a]	-
5a	19.3	829	5	β-D-Glc-(1→4)-β-D-Glc-(1→3)-β-D-Glc-(1→X)-β-D-Glc-(1→X)-D-Glc	2.7	0.4[b]	-

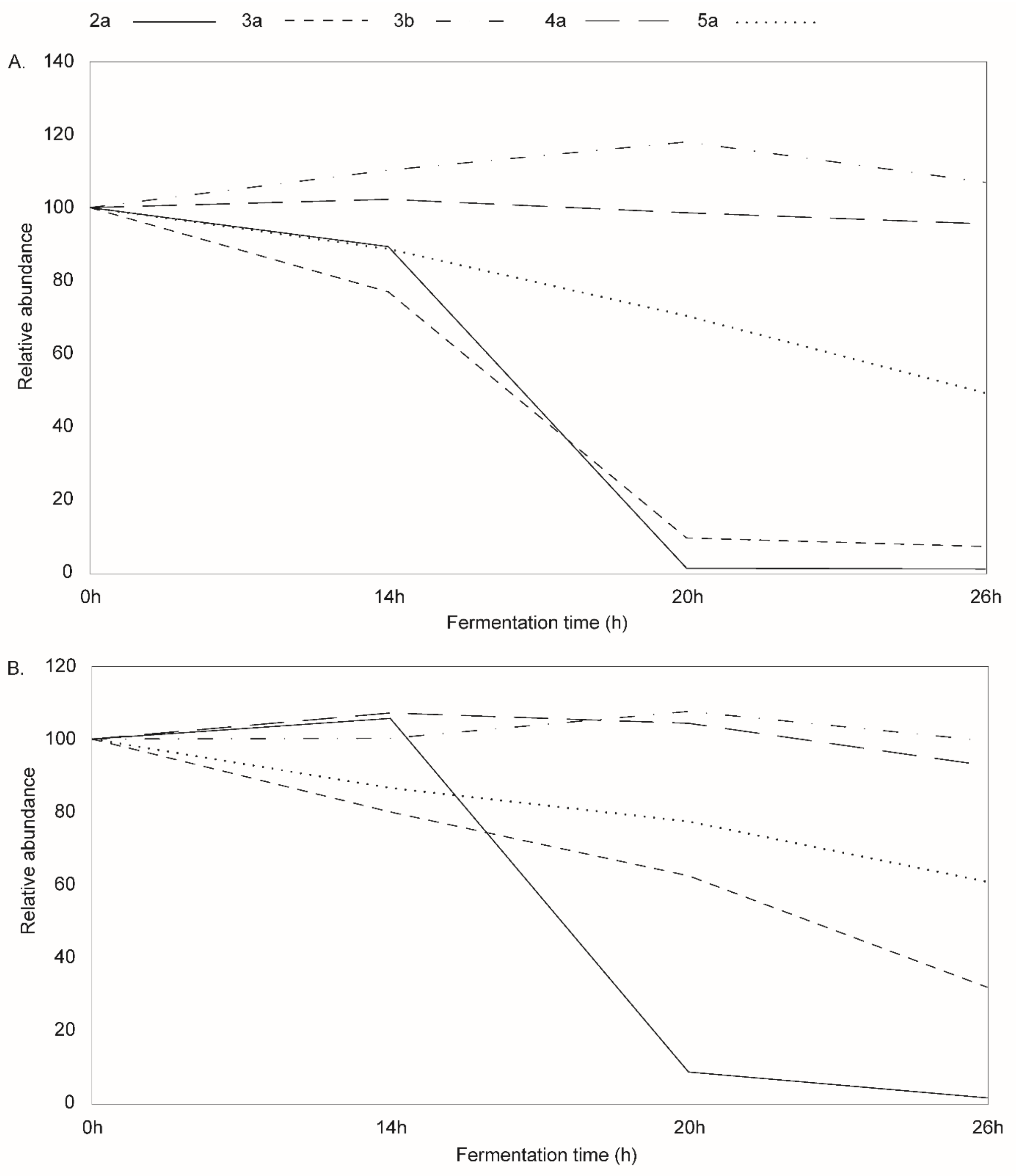

Figure S1. Percentages of remaining enzyme-treated oat β-glucan oligomers during in vitro fermentation using pooled faecal inoculum of 2- (A) and 8- (B) week-old infants. Concentrations per oligomer in the original enzyme-treated oat β-glucan were set at 100%. Chemical structures of oligomer 2a, 3a, 3b, 4a and 5a are summarized in Table S1.

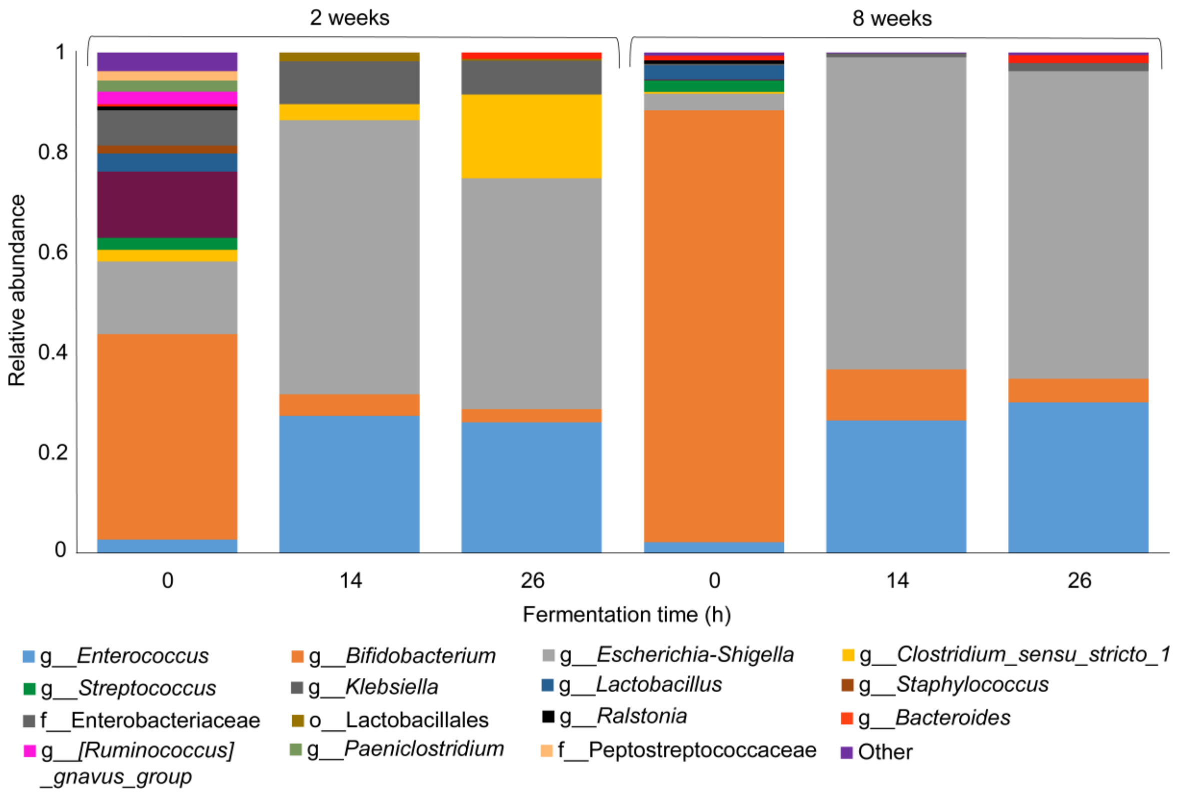

Figure S2. Relative abundance of bacteria at the highest classified taxonomy in fermentation digesta collected at the start and after 14 and 26 h from in vitro fermentation containing solely SIEM medium and faecal inoculum of 2- and 8-week-old infants.

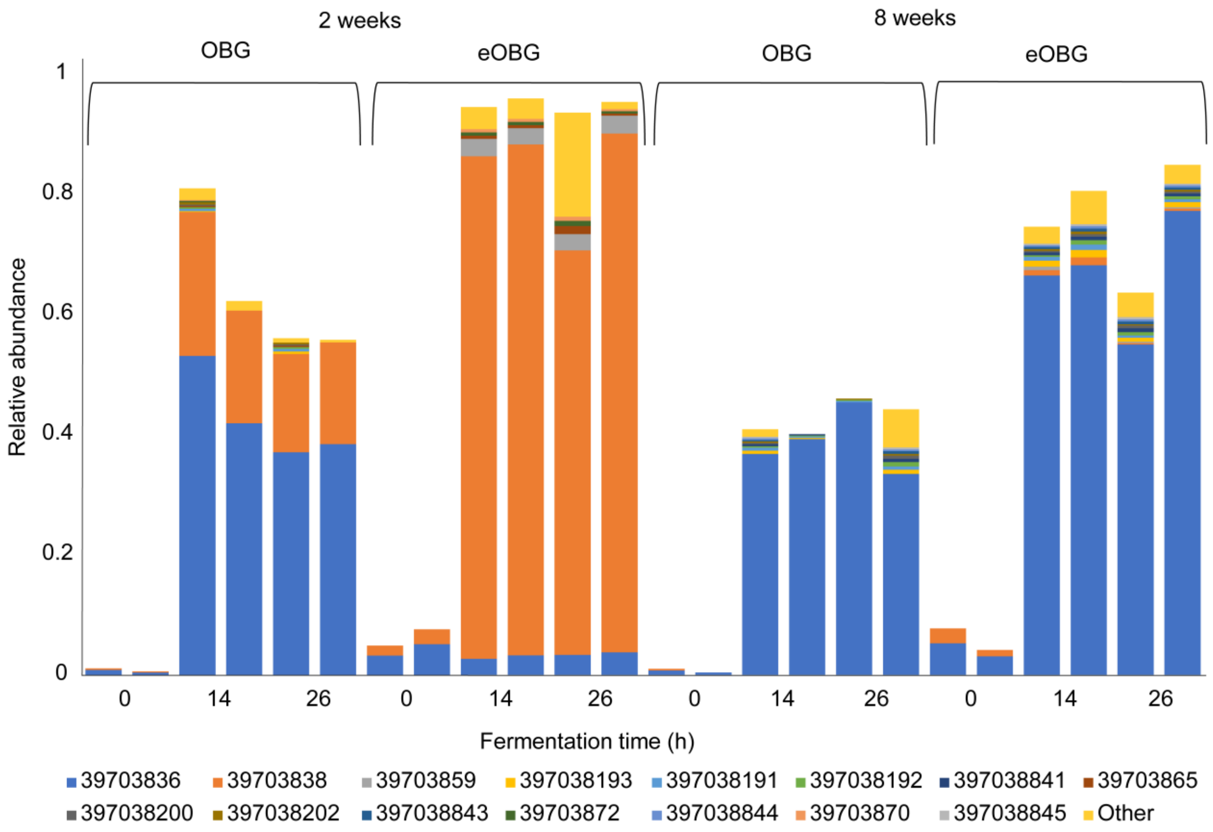

Figure S3. Relative abundance of Enterococcus Amplicon Sequencing Variants in duplicate fermentation digesta collected at the start and after 14 and 26 h from in vitro fermentation of media supplemented with native oat β-glucan (OBG) and enzyme-treated oat β-glucan (eOBG) using pooled faecal inoculum of 2- and 8-week-old infants.

Table S2. Sequences of Enterococcus ASVs 39703836 and 39703838

ASV 39703836
TGGTAGTCCACGCCGTAAACGATGAGTGCTAAGTGTTGGAGGGTTTCCGCCCTTCAGTGCTGCAGCAAAC<br>GTCACTTTGTCCCCGAAGGGAAAGCTCTATCTCTAGAGTGGTCAAAGGATGTCAAGACCTGGTAAGGTTC

ASV 39703838
TGGTAGTCCACGCCGTAAACGATGAGTGCTAAGTGTTGGAGGGTTTCCGCCCTTCAGTGCTGCAGCTAAC<br>GTCACTTTGCCCCCGAAGGGGAAGCTCTATCTCTAGAGTGGTCAAAGGATGTCAAGACCTGGTAAGGTTC

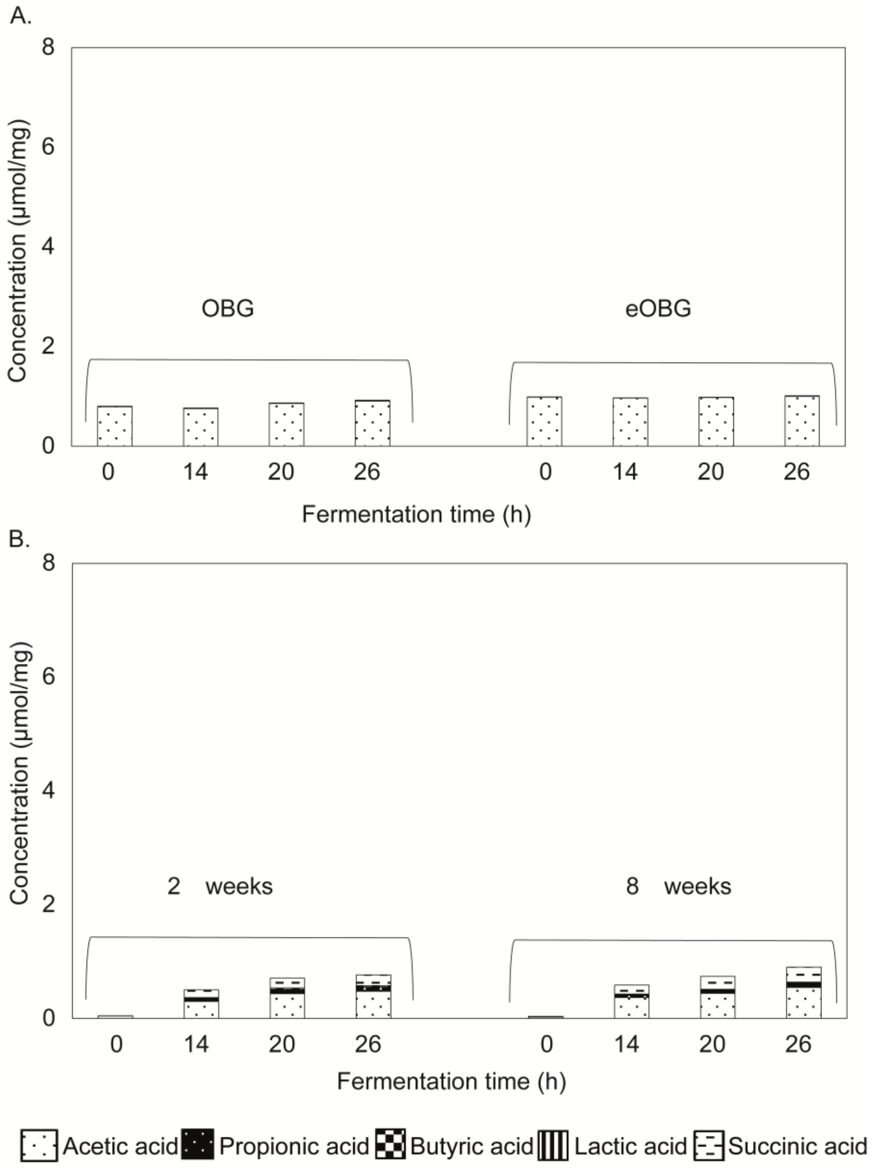

Figure S4. Production of SCFAs, lactic acid and succinic acid upon in vitro fermentation containing solely native oat β-glucan (OBG) / enzyme-treated oat β-glucan (eOBG) and SIEM medium (A) or infant faecal inoculum and SIEM medium (B). The acetate in the control fermentation without added inoculum is primarily originating from the sodium acetate buffer used in the OBG and eOBG preparation.
